# Spontaneous renal allograft rupture complicated by urinary leakage: case report and review of the literature

**DOI:** 10.1186/s12894-015-0109-3

**Published:** 2015-11-18

**Authors:** Evaldo Favi, Samuele Iesari, Alessandro Cina, Franco Citterio

**Affiliations:** Transplant Unit, Renal Department, Royal London Hospital, Whitechapel Road, London, E1 1BB UK; General Surgery, Department of Biotechnological and Applied Clinical Sciences, University of L’Aquila, Via Pompeo Spennati, 67100 L’Aquila, Italy; Department of Bioimaging, Università Cattolica del “Sacro Cuore”, Policlinico Universitario “Agostino Gemelli”, Largo Agostino Gemelli 8, 00168 Rome, Italy; Renal Transplant Unit, Department of Surgery, Università Cattolica del “Sacro Cuore”, Policlinico Universitario “Agostino Gemelli”, Largo Agostino Gemelli 8, 00168 Rome, Italy

**Keywords:** Renal allograft rupture, Urinary leakage, Acute rejection, Graft repair

## Abstract

**Background:**

For more than forty years, graftectomy has been the standard treatment of spontaneous renal transplant rupture. However, recent evidences suggest that graft salvage strategies can be safely pursued, even in difficult cases.

**Case presentation:**

We report on a thirty-nine-year-old woman who received a deceased donor kidney transplant and experienced spontaneous allograft rupture due to acute rejection. The rupture was further complicated by urinary leakage. The kidney and the ureter were successfully repaired. Eight years after transplantation, graft function is still excellent.

**Conclusion:**

Due to the lack of transplantable organs and the long time usually spent on the waiting list, graftectomy should be only considered in case of refractory haemodynamic instability or compromised graft viability.

## Background

Spontaneous renal allograft rupture is a rare but life threatening complication of kidney transplantation. Graftectomy is the safest option. However, if the patient can be stabilised, graft repair should be always considered because of the long waiting list and the low chance of receiving a second transplant.

To the best of our knowledge, this is the first report on a patient who had her transplant saved after experiencing acute rejection, spontaneous graft rupture and urinary leakage.

## Case presentation

A thirty-nine-year-old woman with end stage renal disease secondary to eclampsia was admitted to our institution for her primary deceased donor kidney transplantation on January 2006. Pre-transplant comorbidities included hypertension, dyslipidemia and secondary hyperparathyroism. The donor was a twenty-six-year-old male who died from acute obstruction of the foramina of Monroe. Cold ischemia time was ten hours. The kidney was medium-sized, had one artery, one vein and one ureter. The donor and the recipient were blood group compatible and six HLA antigens mismatched. The highest recipient Panel Reactivity Antibody was 15 % and the direct microcytotoxicity crossmatch was negative. Macroscopically, the kidney looked normal. It was extraperitoneally transplanted in the left iliac fossa, as standard procedure. After declamping, the organ reperfused immediately. The graft ureter was anastomosed to the bladder according to the single stitch technique. The patient was enrolled in a phase III randomized, multicenter, clinical trial and received basiliximab (Simulect®, Novartis Pharmaceuticals Corporation), cyclosporine (Sandimmune Neoral®, Novartis Pharmaceuticals Corporation), everolimus (Certican®, Novartis Pharmaceuticals Corporation) and steroids.

The early post-operative course was complicated by delayed graft function requiring haemodialysis. According to the protocol, her baseline immunosuppression was not changed. Daily ultrasound evaluation showed a well-sized graft with no fluid collections, no hydronephrosis, and good intra-parenchymal flow. Haematologic and coagulation profiles were normal.

**Fig. 1:**
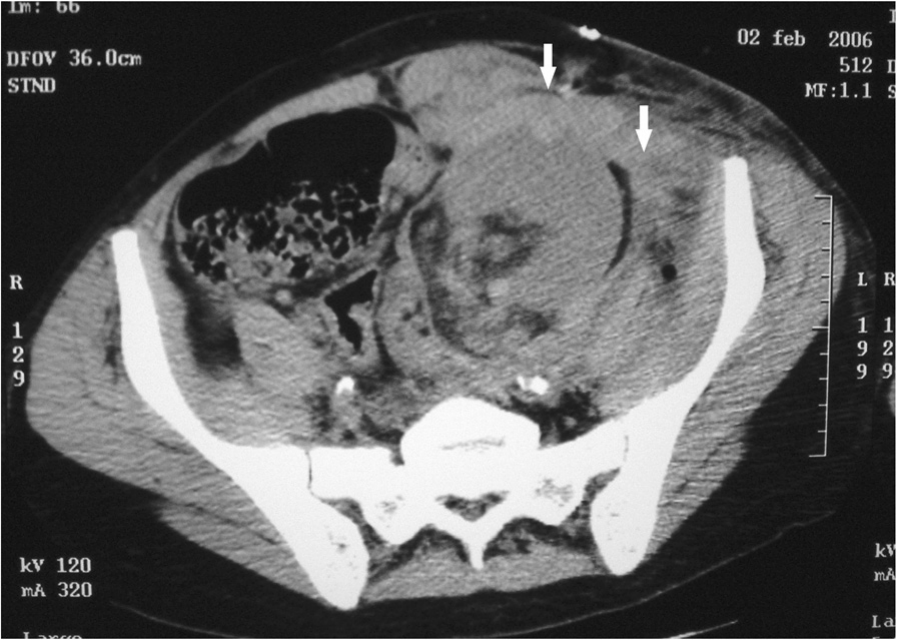
Abdomen and pelvis CT scan showing a massive retroperitoneal haematoma with active bleeding from the transplanted kidney (arrows)

On post-operative day four, the recipient suddenly complained of abdominal pain and the urinary output dropped. Blood pressure, pulse rate and haemoglobin concentration remained stable. Four hours later, the abdominal pain increased and the area over the graft became distended and tender. Hypotension rapidly developed, the pulse rate increased and the patient became anuric. Aggressive resuscitation was promptly initiated. An urgent Doppler ultrasound scan was performed. It showed a large hypoechoic collection surrounding the graft with good flow in the renal artery and a patent renal vein. Subsequent contrast-enhanced CT scan revealed a ruptured graft and a massive retroperitoneal haematoma with active bleeding from the transplanted kidney (Fig. 1). The patient was immediately brought to theatre and explored. A large peri-graft haematoma was evacuated. The kidney was swollen but pink and the arterial pulse was good. A 5 cm long and 1.5 cm deep laceration was found along the middle portion of the convex border of graft, actively bleeding. After en block clamping of the renal vessels, the transplant was fully inspected. Haemostasis was achieved using a haemostatic matrix (Floseal® Hemostatic Matrix, Baxter International Inc.) and by apposition of several mattress polyglactin 910 (Vicryl™, Ethicon Inc.) sutures tied over flaps of absorbable hemostats (Surgicel® Original Absorbable Hemostat, Ethicon Inc.). To prevent extension of the rupture, a polyglactin 910 mesh (Vicryl™ Woven Mesh, Ethicon Inc.) was wrapped around the graft. The procedure took 140 min and renal clamping was 45 min. The total intra-operative blood loss (including the haematoma) was 500 mL and two units of packed red blood cells were transfused. Considering the severity of the bleeding, the complex repair, and the risk of further damage to the kidney, a graft biopsy was not taken at the time of the operation. However, the clinical picture was highly suggestive of acute rejection and we therefore decided to administer a course of rabbit anti-thymocyte globulins (Thymoglobulin®, Genzyme Inc.). Cyclosporine and everolimus were also switched to tacrolimus (Prograf®, Astellas Pharma Inc.) and mycophenolate mofetil (CellCept®, Genentech USA Inc.). After the operation, the patient remained anuric requiring haemodialysis for twenty-one days. Renal function improved and serum creatinine concentration fell to 2 mg/dL.

**Fig. 2:**
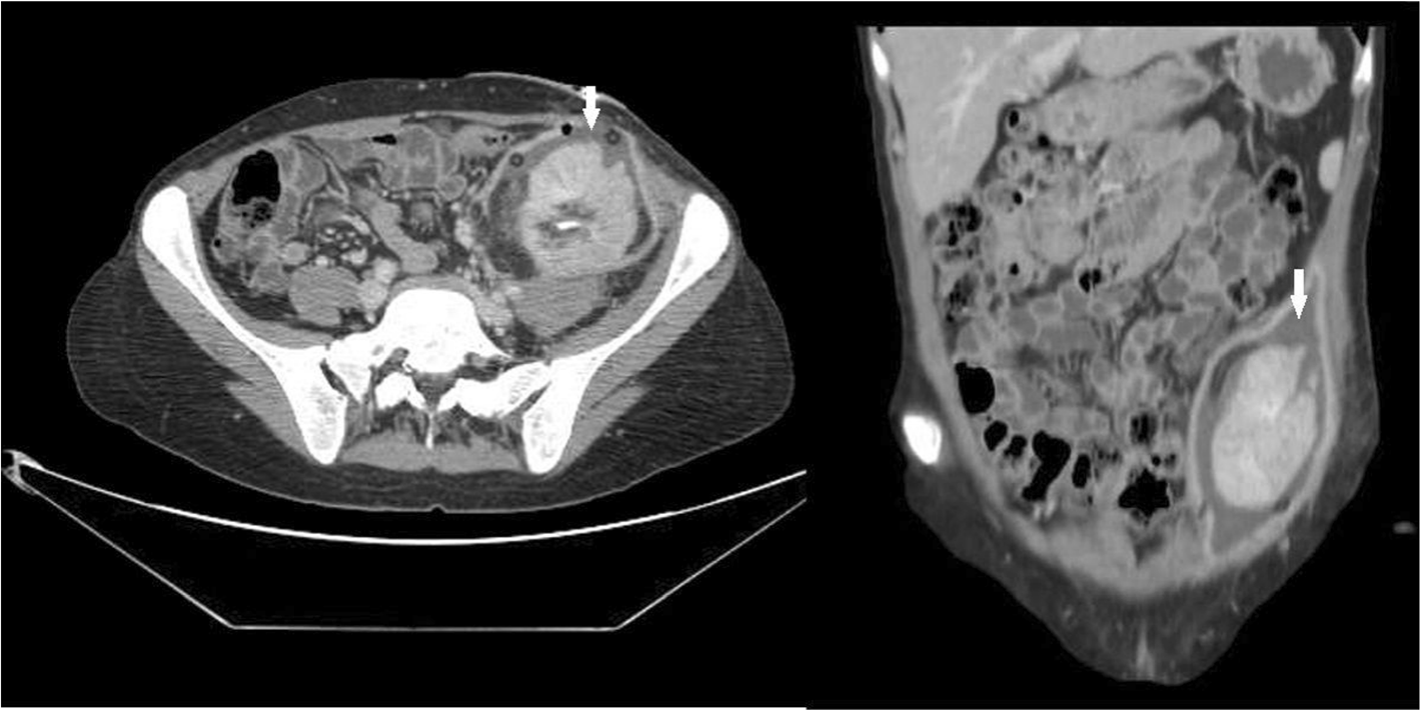
Abdomen and pelvis CT scan showing a large urine collection around the graft, in continuity with the area of the previous rupture (arrows). (left) axial reconstruction; (right) coronal reconstruction

**Fig. 3:**
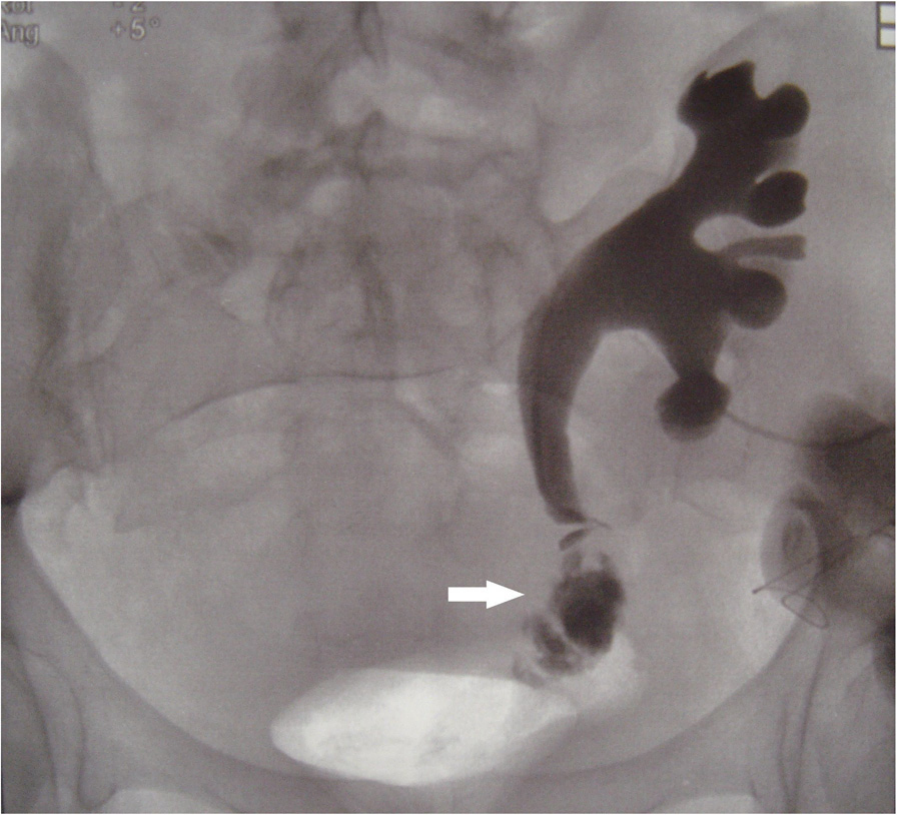
Nephrostogram demonstrating urinary leakage at the site of the ureteral reimplantation (arrow)

On post-operative day fifty-two, clear fluid started to seep from the wound. Urgent CT scan showed a large (5 × 2.3 cm) fluid collection in continuity with the area of the previous rupture (Fig. 2). Urinary leakage was confirmed by chemical analysis of the fluid. A nephrostomy tube and a trans-abdominal drain were inserted to control the leak and evacuate the collection. Two days later, the patient became febrile and the urinary output dropped. A nephrostogram was performed. It demonstrated another urinary leak at the site of the ureteral anastomosis (Fig. 3). Following an unsuccessful simultaneous cystoscopy and fluoroscopy guided ureteral stent placement, a surgical exploration was planned. The abdomen was entered through a midline incision and the graft was inspected. The distal part of the transplanted ureter, partially necrotized, was resected and the proximal stump was anastomosed to the bladder according to the Lich-Gregoire technique. A double-J pyeloureteral stent was placed to protect the anastomosis. The procedure took 143 min. The post-operative course was uneventful. Renal function gradually recovered, serum creatinine fell to 1.7 mg/dL and the patient was eventually discharged.

Eight years after transplantation, the patient is doing well and her serum creatinine is 1 mg/dL.

## Discussion

Spontaneous renal allograft rupture is defined as a laceration of the renal capsule when there are no other identifiable injuries noted at the time of the organ retrieval [1]. Its incidence has been reported as between 0.3 and 9.6 %, depending on the series [2–4]. This complication usually occurs within the first two weeks after transplantation although late ruptures have been reported [5].

Several causes have been proposed [1–3, 5–7]. Acute rejection is the most important, accounting for 60 to 80 % of cases. Less frequent etiologies include renal vein thrombosis, acute tubular necrosis, ureteral obstruction, renal biopsy, trauma, local ischaemia, septic infarction and cancer. The rupture most frequently occurs longitudinally, along the convex border of the kidney. Clinical presentation is often typical, with severe graft dysfunction and acute blood loss. Immediate ultrasound evaluation can rapidly and safely confirm the diagnosis with 87 % sensitivity and 100 % specificity [8].

In case of haemodynamic instability, immediate surgical exploration is mandatory. Initial reports of conservative management of graft rupture showed poor results, with less than 30 % success rate. Failure to control acute bleeding, inability to reverse acute rejection, post-surgical development of multiple organ failure and uncontrollable coagulopathy were the main causes of the high rate of graft nephrectomies observed. In the last decades, improved surgical technique and post-operative care have significantly reduced transplant-related mortality and morbidity. Complex operations are now safely performed with good results. Current reports demonstrate that ruptured grafts can be saved with a success rate as high as 80 %. Moreover, recipients undergoing successful repair have long-term outcomes similar to the general transplant population. Recurrent rupture, the most dangerous complication of graft repair, only occurs in 5 % of patients and does not significantly jeopardise the prognosis (see Table 1 for details). Fibrin glue and collagen foam can be used to facilitate haemostasis without endangering the transplant through unnecessary manipulation of oedematous and fragile tissues [9]. Renal corsetage with various materials, including polyglactin 910 mesh, lyophilized dura, grafts of peritoneum, pieces of external oblique aponeurosis and polypropylene mesh has also been reported [10]. In this setting, external compression is particularly helpful because it supports haemostasis and at the same time prevents further extension of the rupture.

**Table 1:** Management and outcomes of spontaneous renal allograft rupture (SRAR) over time

Authors year	SRAR/KTx (#)	Incidence (%)	p.o. day mean	p.o. day median	p.o. day range	FU MAX Months	Graftectomy (# / %)	Repair (# / %)	Death (# / %)
Murray 1968 [11]	4/110	3.6	3 ± 2	2.5	1 - 6	33	1 / 33 %	3 / 77 %	0
Salaman 1969 [12]	3/74	4.1	-	-	-	-	2 / 66.6 %	1 / 33.3 %	-
Siedek 1969 [13]	1/21	4.8	9	9	-	-	1 / 100 %	0	0
Flanigan 1971 [14]	2/46	4.3	-	-	≤8	-	2 / 100 %	0	0
Haimov 1971 [15]	1/30	3.3	2	2	2	4	0	1 / 100 %	0
Lord 1972 [16]	1/280	0.4	14	-	-	0.5	1 / 100 %	0	1 / 100 %
Minale 1972 [17]	6/100	6	4.5 ± 1	4	3 - 7	60	0	6 / 100 %	0
Ghose 1973 [18]	6/71	8.4	-	-	-	-	3 / 50 %	3 / 50 %	-
Fjeldborg 1974 [19]	7/200	3.5	-	-	-	-	2 / 28.6 %	5 / 71.4 %	-
Kootstra 1974 [20]	2/39	5.1	27 ± 27	27	8 - 46	-	1 / 50 %	0	1 / 100 %
Homan 1977 [21]	21/246	8.5	-	-	2 - 49	8	2 / 9.5 %	19 / 90.5 %	0
Van Cangh 1977 [22]	9/325	2.8	-	-	-	-	6 / 66.6 %	3 / 33.3 %	-
Brekke 1978 [23]	16/448	3.6	-	-	-	-	10 / 62.5 %	6 / 37.5 %	-
Montes 1978 [24]	13/419	3.1	-	-	-	-	5 / 38.5 %	8 / 61.5 %	-
Susan 1978 [25]	4/474	0.8	10.5 ± 5	10.5	5–16	15	0	4 100 %	0
Dryburgh 1979 [26]	9/93	9.7	-	-	1–18	22	7 / 78 %	2 / 22 %	2 / 22 %
Prompt 1979 [27]	8/327	2.4	7 ± 3.5	8.5	2–11	0.8	6 / 75 %	2 / 25 %	0
Oesterwitz 1980 [28]	22/364	6	5 ± 3	4.5	1–14	60	10 / 45 %	12 / 55 %	0
Van Der Vliet 1980 [29]	1/211	0.5	-	-	-	-	1 / 100 %	0	-
Goldman 1981 [30]	7/350	2	-	-	3–7	58	3 / 43 %	4 / 57 %	0
Nghiem 1981 [31]	7/585	1.2	18 ± 20	8	3–58	96	2 / 29 %	5 / 71 %	0
Thukral 1982 [32]	3/100	3	7 ± 1	8	6–8	60	0	3 / 100 %	0
Serrallach 1985 [33]	5/66	7.6	7 ± 3	8	4–10	15	0	5 / 100 %	0
Chopin 1989 [34]	4/85	4.7	17 ± 13	15	5–32	12	1 / 25 %	3 / 75 %	0
Said 1994 [10]	3/75	4	7 ± 3.5	7	4–11	10	2 / 67 %	1 / 33 %	0
Yadav 1994 [35]	15/237	6.3	-	-	-	120	4 / 27 %	11 / 73 %	-
Heimbach 1995 [4]	8/238	3.4	11	-	8–17	94	1 / 12.5 %	7 / 87.5 %	0
Azar 1996 [3]	12/331	3.6	10	9	4–21	-	12 / 100 %	0	1 / 8 %
Zadrozny 1997 [36]	8/112	7.1	-	-	-	-	5 / 62.5 %	3 / 37.5 %	0
Pontones Moreno 1998 [37]	21/868	2.4	-	-	-	-	4 / 19 %	17 / 81 %	0
Szenohradszky 1999 [7]	53/628	8.4	-	-	-	-	37 / 70 %	16 / 30 %	-
Millwala 2000 [38]	4/145	2.7	-	-	-	6	2 / 50 %	2 / 50 %	1 / 25 %
Ramos 2000 [39]	11/934	1.2	5	-	2–13	-	10 / 90.9 %	1 / 9.1 %	0
Hochleitner 2001 [2]	14/1811	0.8	111 ± 6	9.5	3–23	111	5 / 36 %	9 / 64 %	0
Guleria 2003 [40]	3/172	1.7	6 ± 1	7	5–7	-	0	3 / 100 %	0
Finley 2003 [5]	22/4418	0.5	299 ± 793	7	0–2825	214	8 / 36 %	14 / 64 %	0
He 2003 [41]	38/1000	3.8	-	-	-	60	2 / 5.3 %	31 / 81.6 %	0
Busi 2004 [42]	4/778	0.5	77.5 ± 4	6.5	4–13	0.5	3 / 75 %	1 / 25 %	0
Risaliti 2004 [43]	2/297	0.7	-	-	-	-	-	-	-
Sanchez de la Nieta 2004 [44]	10/657	1.5	7.5	-	1–10	-	5 / 50 %	5 / 50 %	0
Shahrokh 2005 [45]	6/1682	0.4	6	-	4–13	60	3 / 50 %	3 / 50 %	0
Martinez Mansur 2006 [46]	11/492	2.8	-	-	-	-	7 / 63.6 %	4 / 36.4 %	-
Overall	407/19939	2	-	-	0–2825	53	176 / 44.2 %	223 / 54.8 %	7 / 1.8 %

## Conclusions

When spontaneous renal transplant rupture occurs, nephrectomy is justified only in case of refractory haemodynamic instability or compromised kidney viability. When irreversible graft damage can be ruled out and the patient can be readily resuscitated, transplant salvage should always be attempted.

## Consent

Written informed consent for publication of this Case report and any accompanying images has been obtained from the patient. A copy of the written consent is available for review by the Editor of this journal.

## Abbreviation

CT: Computed tomography
